# Death of Dr. Hunt

**Published:** 1867-08

**Authors:** 


### *Death of Dr. Hunt.*

Our gifted, amiable and excellent contributor, R. P. HUNT, M.D., has met with sudden death. It is supposed that while sitting at an open window in his office, he dropped asleep, and fell into the area below. The accident was not discovered until the following morning. Dr. H. was a gentleman of rare culture, extensive observation, and large professional acquirements. Our own acquaintance had been brief, but sufficient to enable us to fully accept the testimonial to his memory, which we extract from the Louisville *Journal:*

"Many of our citizens were startled yesterday by the sad news of the sudden death of Dr. R. P. Hunt, in Chicago, where he had been sojourning for some months. He was well known to our people—to many personally, and to all by reputation.

"Dr. Hunt was born in Lexington. He was of one of the best families in the country—best in the best sense of the word best. He received a fine classical and medical education, but, possessing large wealth, he was never, we believe, a candidate for medical practice. He devoted much time to literature, to solid reading, and to the pleasures of friendship. He was one of the most delightful and fascinating of companions. He had all the social qualities in an eminent degree, and shone in every high circle. He was open, sincere, just, generous, warm-hearted, and chivalric. Toward all, of every respectable position, he always exhibited the most knightly courtesy. He was a gentleman by nature and education, polished to the highest degree of refinement and elegance by constant intercourse with the best portion of the world. His manners were those of a Paladin, and his thoughts and feelings corresponded with them. He was noble without being aristocratic. He was charitable to faults, but his scorn of all things mean and low was, although calm, intensely bitter.

"Dr. Hunt leaves a wife and child, the latter the lovely and delicate bud of an immortal flower, and the former known throughout all the country for her beauty, her goodness, and her accomplishments. Alas, that the sympathies of friends have so little power to soothe the distressed."

### *Obituary of Dr. Geo. K. Amerman.*

Notice of the death of Dr. Amerman, resolutions of respect, condolence, etc., were in type for this No., but excess of matter has crowded the article out in making up the forms. Will appear in next JOURNAL.

